# Efficiency of the Inclusion of Rebamipide in the Eradication Therapy for *Helicobacter pylori* Infection: Meta-Analysis of Randomized Controlled Studies

**DOI:** 10.3390/jcm8091498

**Published:** 2019-09-19

**Authors:** Dmitrii N. Andreev, Igor V. Maev, Diana T. Dicheva

**Affiliations:** Moscow State University of Medicine and Dentistry named after A.I. Evdokimov, 127473 Moscow, Russia; igormaev@rambler.ru (I.V.M.); di.di4eva@yandex.ru (D.T.D.)

**Keywords:** *Helicobacter pylori*, therapy, eradication, rebamipide

## Abstract

Background: There has been a negative trend in the effectiveness of classic eradication therapy regimens for *Helicobacter pylori* (*H. pylori*), which has largely been determined from the emergence and spread of antibiotic resistance. Several studies have shown that adding rebamipide to eradication regimens leads to an increase in the effectiveness of treatment. Aim: To evaluate the efficacy and safety of including rebamipide in the eradication regimens for *H. pylori* infection. Methods: The literature search was conducted in the MEDLINE/PubMed, EMBASE, Cochrane Central Register, Korean Medical Citation Index, and Russian Science Citation Index databases. All identified randomized controlled trials comparing rebamipide supplementation with non-rebamipide-containing eradication regimens for the treatment of *H. pylori* infection were included in the final analysis. Results: We identified 11 randomized controlled trials (RCTs) involving 1227 patients (631 in groups with rebamipide and 596 in groups without rebamipide). The meta-analysis showed that the addition of rebamipide to eradication regimens significantly increased the effectiveness of treatment (odds ratio (OR) 1.753, 95% confidence interval (CI) 1.312–2.333, *p* < 0.001). The subgroup analysis demonstrated that rebamipide significantly increased the effectiveness of eradication when added to a dual therapy regimen (OR 1.766, 95% CI: 1.167–2.495, *p* = 0.006); however, no significant improvement in effectiveness was observed when it was added to the triple therapy regimen (OR 1.638, 95% CI 0.833–3.219, *p* = 0.152). Conclusion: This meta-analysis demonstrated that the addition of rebamipide to *H. pylori* eradication regimens significantly increases the effectiveness of treatment.

## 1. Introduction

*Helicobacter pylori* (*H. pylori*) is one of the most common human pathogens [1]. According to the latest systematic review, approximately 45.4% of the world’s population is infected with this microorganism [2]. *H. pylori* infection is a leading etiological factor for various gastroduodenal diseases, including chronic gastritis, peptic ulcers and duodenal ulcers, as well as adenocarcinoma and MALT lymphoma of the stomach [1,3,4].

According to the latest European (Maastricht V, 2015) [5] and North American (Toronto, 2016; American College of Gastroenterology, 2017) [6,7] recommendations for the diagnosis and treatment of *H. pylori* infection, eradication therapy should be administered to all infected people. Such tactics can achieve resolution of inflammatory changes in the gastric mucosa and prevent the development of precancerous conditions (atrophic gastritis, intestinal metaplasia) [8,9,10]. However, in the last decade, there has been a negative trend in the effectiveness of classic eradication therapy regimens, which has largely been determined by the emergence and spread of antibiotic resistance [3,11]. According to the latest meta-analyses, the effectiveness of one of the eradication schemes most commonly used in clinical practice—triple therapy—is at a relatively low level (approximately 69–77%) [12,13,14]. Given the lack of fundamentally new drugs for the treatment of *H. pylori* infection, it is important to optimize the existing eradication regimens [15,16]. In this regard, promising results were demonstrated when bismuth [17,18] or probiotics [19,20] were added to eradication regimens. In addition, the addition of the gastroprotective drug rebamipide to eradication regimens has shown great potential [21,22].

The principal mechanisms of action of rebamipide are the induction of prostaglandin synthesis in the gastric mucosa, neutralization of oxidative stress products, and inhibition of neutrophil activation [22]. Rebamipide does not have a direct anti-helicobacter action; however, in experimental studies, it was shown that it inhibits the adhesion of *H. pylori* to epithelial cells of the gastric mucosa [23] and reduces the activation of NF-кB and IL-8 production induced by *H. pylori* [24]. The meta-analysis by Nishizawa et al. [25] summarized the results of six randomized controlled trials (RCTs) and found that the inclusion of rebamipide in eradication therapy significantly increased the effectiveness of treatment (odds ratio (OR) 1.737, 95% confidence interval (CI) 1.194–2.527, *p* = 0.0049). However, all studies included in that meta-analysis were conducted in Asian populations. Moreover, in most studies, the effectiveness of the inclusion of rebamipide was studied in the context of dual therapy, which is currently rarely used in clinical practice. The main purpose of this meta-analysis is to update the data on the effect of rebamipide on the effectiveness of eradication therapy for *H. pylori* infection.

## 2. Materials and Methods

### 2.1. Study Sources and Search

The literature search was conducted in the MEDLINE/PubMed, EMBASE, Cochrane Central Register, Korean Medical Citation Index, and Russian Science Citation Index databases until April 2019. In the above databases, we analyzed the titles, abstracts and keywords. The search used the following combination of keywords: “rebamipide” and “*Helicobacter*” or “*Helicobacter pylori*” or “*H. pylori*”. In the case of one publication duplicated in different databases, only one was selected for the final analysis.

### 2.2. Study Selection

All studies that were identified by the literature search were reviewed and selected according to the following a priori criteria: RCTs with at least two comparison groups; the administration of rebamipide simultaneously with the administration of the eradication regimen; the determination of the primary diagnosis and subsequent eradication with validated tests (C13 urea breath test, rapid urease test, and histological or cultural examinations); and the accomplishment of eradication no earlier than 4 weeks after the end of the course of eradication therapy. 

Included studies were also assessed using the Cochrane risk-of-bias tool. We considered the following domains when assessing the risk of bias of included RCTs: selection bias (random sequence generation and allocation concealment); performance bias (blinding of participants and personnel); detection bias (blinding of outcome assessment); attrition bias (incomplete outcome data); reporting bias (selective outcome reporting); and other sources of bias.

### 2.3. Statistical Analysis

Statistical data processing was performed with MedCalc (version 18.5, MedCalc Software, Ostend, Belgium) and Comprehensive Meta-Analysis (version 3.3.070, Biostat, Chicago, IL, USA) in Microsoft Windows 10 (version 1709, Microsoft, Redmond, WA, USA). The results are presented as ORs and 95% CIs for the efficacy of eradication with rebamipide-containing eradication therapy regimens compared to the efficacy of those without rebamipide. The heterogeneity among the various studies was assessed using Cochrane’s Q test and the I^2^ statistic. Substantial heterogeneity was indicated by *p* < 0.05 and I^2^ > 50. The probability of publication bias existing was estimated by the visual inspection of a funnel plot and the calculation of the Begg–Mazumdar correlation test and Egger’s test.

## 3. Results

### 3.1. Description of the Studies

**Figure 1:**
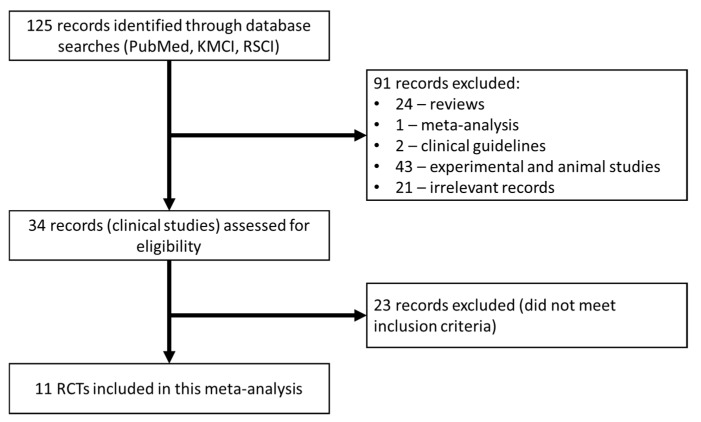
CONSORT chart detailing the study selection strategy.

**Figure 2:**
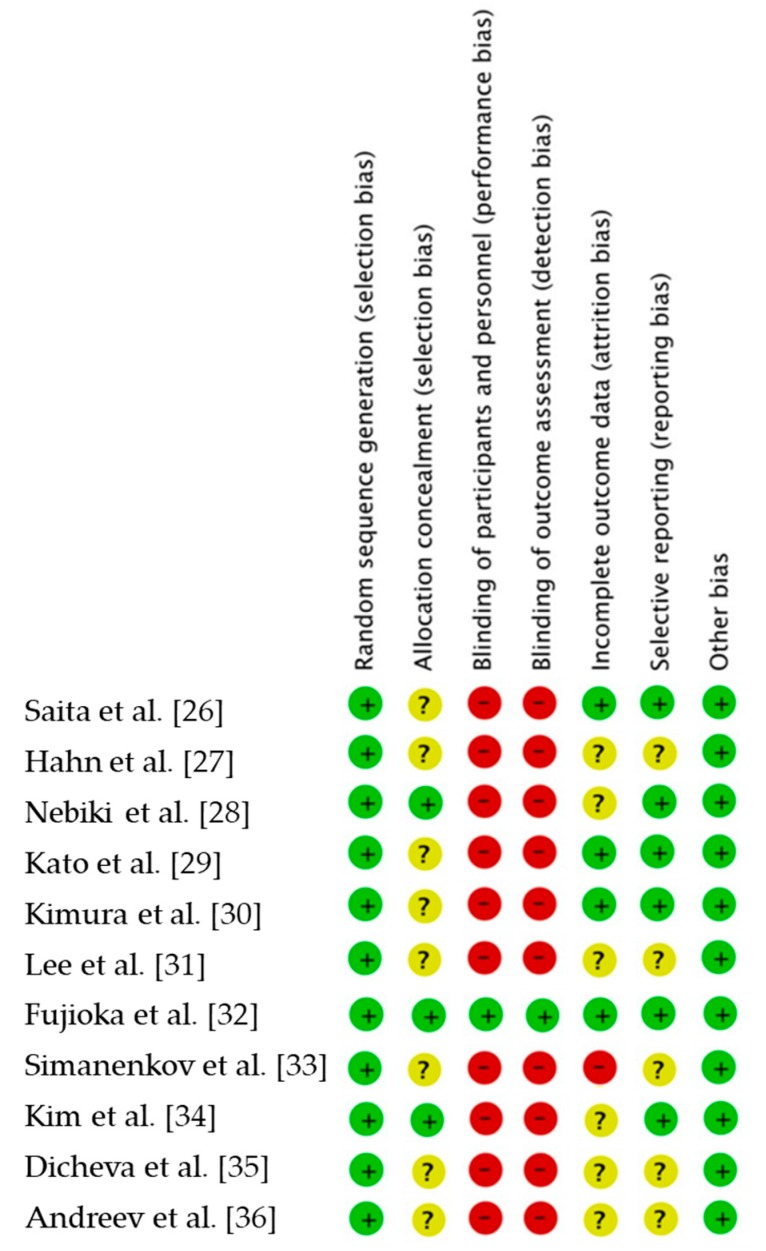
Risk of bias. Green, low risk of bias; yellow, unclear risk of bias; red, high risk of bias.

The database search initially identified 125 papers that were then further analyzed. Of these, 91 studies were excluded because they were not original clinical studies (24—reviews; 43— experimental studies; 2—clinical recommendations; 1—meta-analysis; 21—other irrelevant works). The 34 selected papers were analyzed in detail for their adherence to the inclusion criteria, after which 23 studies were excluded (Figure 1). As a result, 11 studies were included in this meta-analysis (Table 1) [26,27,28,29,30,31,32,33,34,35,36]. The risk of bias in the RCTs is shown in Figure 2. Only one trial was assessed as having a low risk of bias across all domains.

### 3.2. Eradication Rates

**Figure 3:**
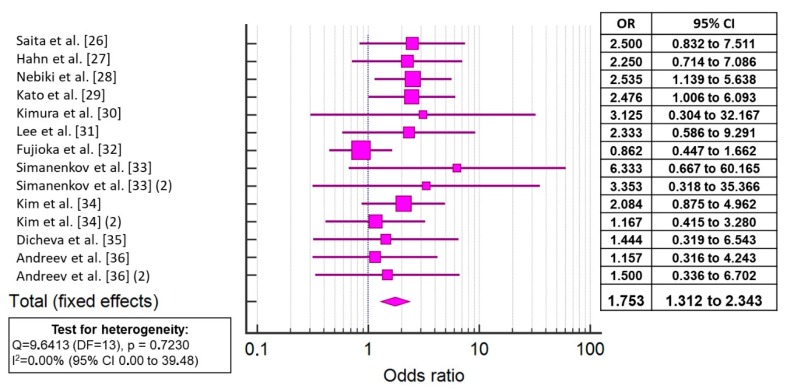
Forest plot showing the odds rations (ORs) and 95% CIs for the effectiveness of the inclusion of rebamipide in the eradication treatment regimen for *Helicobacter pylori* infection.

In the 11 RCTs involving 1227 patients (631 in groups with rebamipide; 596 in groups without rebamipide), the overall eradication efficacy levels were 82.72% in patients taking rebamipide and 73.99% in patients who received the eradication regimen without rebamipide. The meta-analysis showed that the addition of rebamipide to the eradication regimens significantly increased the effectiveness of treatment (OR 1.753, 95% CI 1.312–2.333, *p* < 0.001) (Figure 3, Supplementary Table S1). There was no significant heterogeneity among the studies (*p* = 0.723; I^2^ = 0.00%); therefore, a fixed effects model was used in the subsequent analysis. For additional verification of the obtained results, we excluded from the analysis the two papers with the largest CIs [30,33] and obtained approximately the same results (OR 1.633, 95% CI: 1.234–2.242, *p* = 0.001).

**Figure 4:**
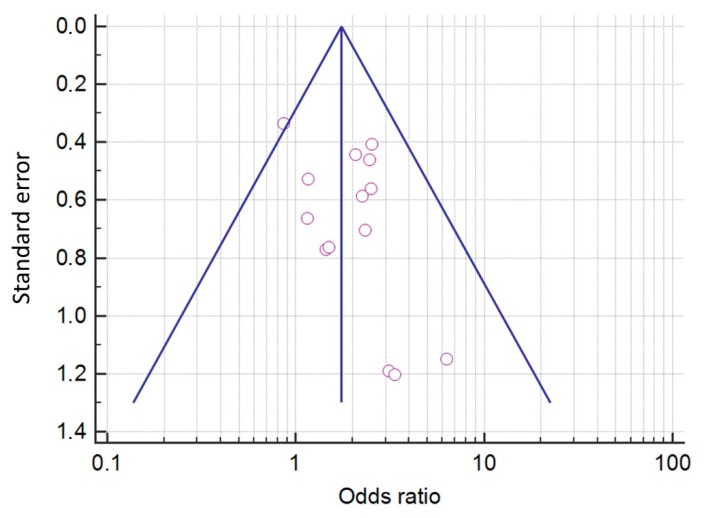
A funnel plot estimating the likelihood of a publication bias.

The probability of the existence of a publication bias was estimated by the visual inspection of a funnel plot and the calculation of the Begg–Mazumdar and Egger’s tests. Visual analysis of the funnel plot (Figure 4) revealed no pronounced asymmetry. In addition, a significant publication bias was not indicated by the results of the Begg–Mazumdar test (Kendall’s tau *b*—0.08791; *p* = 0.33071) or Egger’s test (*p* = 0.09975).

### 3.3. Subgroup Analysis of the Efficacy of Eradication

#### 3.3.1. Duration of Rebamipide Use 

**Figure 5:**
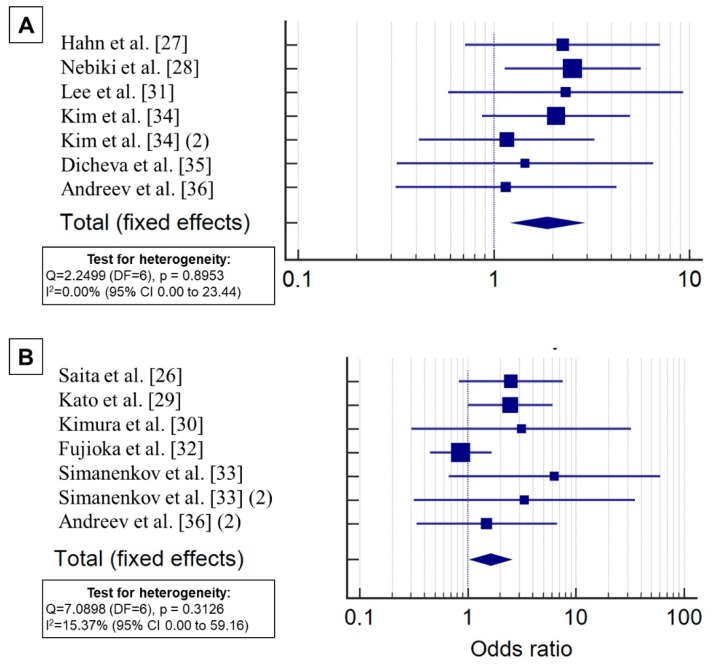
Subgroup analysis of the effect of rebamipide on the efficacy of eradication therapy for *H. pylori* infection with short (**A**) and long (**B**) courses of use.

In five RCTs, rebamipide was administered for a short period ranging from 10 days to 2 weeks (parallel to the beginning and end of the administration of the eradication regimen); in the other five RCTs, rebamipide was administered for a long period ranging from 4 to 12 weeks (parallel to the start of the use of the eradication regimen with subsequent prolongation after finishing the course of antibiotic therapy); and in one RCT, one group received rebamipide for a short period, and the other received rebamipide for a longer period (Table 2). The subgroup analysis demonstrated that rebamipide significantly increased the efficiency of eradication when administered for both short periods (OR 1.880, 95% CI: 1.258–2.808, *p* = 0.002) and long periods (OR 1.625, 1.069–2.471, *p* = 0.023) (Figure 5). No significant heterogeneity among the study results in the subgroups was found (*p* = 0.8953, I^2^ = 0.00% and *p* = 0.3126, I^2^ = 15.37%).

#### 3.3.2. The Eradication Regimen with the Inclusion of Rebamipide 

In five RCTs, rebamipide was added to the dual eradication therapy regimen, in four RCTs it was added to the triple therapy, and in two other RCTs it was added to the triple therapy with bismuth preparation and to the concomitant therapy (Table 3). The subgroup analysis demonstrated that rebamipide significantly increased the effectiveness of eradication when added to a dual therapy regimen (OR 1.766, 95% CI: 1.167–2.495, *p* = 0.006); however, no significant improvement in effectiveness was observed when it was added to the triple therapy regimen (OR 1.638, 95% CI 0.833–3.219, *p* = 0.152).

#### 3.3.3. Ethnicity

Of the 11 studies included in this meta-analysis of RCTs, eight studies were conducted with Asian patient populations (5—Japan; 3—South Korea), and the other three were conducted with Caucasoid populations (all—Russia). The inclusion of rebamipide in eradication therapy regimens significantly increased the efficacy of treatment in individuals in the Asian populations (OR 1.742, 95% CI: 1.268–2.391, *p* = 0.001), but there was no significant effect in the Caucasoid populations (OR 1.882, 95% CI: 0.888–3.700, *p* = 0.103).

#### 3.3.4. Side Effects

Among the 11 RCTs, data on the incidence of side effects during therapy were available in only five papers. A meta-analysis of the frequency of adverse events did not reveal significant differences between the groups that did or did not receive rebamipide (OR 1.279, 95% 0.915–1.789, *p* = 0.150).

## 4. Discussion

*H. pylori* is a common human bacterial infection and the leading cause of chronic gastritis, gastric and duodenal ulcers and adenocarcinoma and MALT lymphoma of the stomach [1,3,4]. Traditionally, in clinical practice, the combination of PPIs and antibacterial drugs is used to eradicate *H. pylori* [11]. However, as shown by recent major studies, the frequency of treatment failure when using this combination is approximately 20–30% [15]. This is largely determined by the increase in the number of resistant strains of *H. pylori* in the population [11,37]. Given the lack of fundamentally new drugs for the treatment of *H. pylori* infection, it is important to optimize the existing eradication schemes [15,16]. The inclusion of rebamipide in eradication therapy regimens seems to be quite promising. This drug does not have its own direct anti-helicobacter action; however, in experimental studies, it was shown to inhibit the adhesion of *H. pylori* to epithelial cells of the gastric mucosa and to have an anti-inflammatory effect by reducing the production of IL-8 induced by *H. pylori* [22,23,24].

This meta-analysis of 11 RCTs demonstrated that the addition of rebamipide to eradication regimens significantly increases the effectiveness of treatment (OR 1.753, 95% CI 1.312–2.343, *p* < 0.001). The data obtained are nearly comparable with the results of the early meta-analysis by Nishizawa et al. [25], which analyzed studies from Asian countries (OR 1.737, 95% CI 1.194–2.527, *p* = 0.0049).

The subgroup analysis showed that rebamipide significantly increases the efficiency of eradication when used for either a short period (10 days–2 weeks) or a prolonged period (4–12 weeks). It seems that the long-term use of rebamipide in the post-eradication period is justified because several studies with long observation periods have shown that the drug is effective at reducing the inflammatory changes in the gastric mucosa [32,38].

There are several limitations in this meta-analysis. In particular, the RCTs included in the analysis were conducted in only three countries (Japan, South Korea, Russia). In addition, there is substantial heterogeneity among the included studies, stemming from the different methods of diagnosing and controlling the eradication of *H. pylori* infection and the different durations and dosages of the drugs prescribed. Despite the fact that the present work showed that rebamipide significantly increases the effectiveness of eradication when added to a dual therapy scheme, there was no significant effect when it was added to a triple therapy regimen, which is explained by the scarcity of data. To confirm the role of rebamipide in increasing the effectiveness of eradication therapy for *H. pylori* infections, further larger RCTs are needed that are conducted in a substantial number of regions worldwide.

## 5. Conclusions

Thus, this meta-analysis demonstrated that the addition of rebamipide to *H. pylori* eradication regimens significantly increases the effectiveness of treatment.

## Figures and Tables

**Table 1:** Characteristics of the selected studies.

Study, Year	Country	Rebamipide-Containing Regimen(s)	Comparison Regimen(s)
Saita et al. [26]	Japan	Dual therapy—2 weeks (w); rebamipide—8 w	Dual therapy—2 w
Hahm et al. [27]	Korea	Dual therapy—2 w; rebamipide—2 w	Dual therapy—2 w
Nebiki et al. [28]	Japan	Dual therapy—2 w; rebamipide—2 w	Dual therapy—2 w
Kato et al. [29]	Japan	Dual therapy—2 w; rebamipide—8 w	Dual therapy—2 w; teprenone—8 w
Kimura et al. [30]	Japan	Triple therapy—1 w; rebamipide—12 w	Triple therapy—1 w; teprenone—12 w
Lee et al. [31]	Korea	Triple therapy—2 w; rebamipide—2 w	Triple therapy—2 w
Fujioka et al. [32]	Japan	Dual therapy—2 w; rebamipide—8 w	Dual therapy—2 w
Simanenkov et al. [33]	Russia	Triple therapy with bismuth—10 days (d); rebamipide—4 w	Triple therapy—10 d
			Triple therapy with bismuth—10 d
Kim et al. [34]	Korea	Concomitant therapy—10 d; rebamipide—10 d	Concomitant therapy—10 d
			Concomitant therapy—10 d; ecabet—10 d
Dicheva et al. [35]	Russia	Triple therapy—10 d; rebamipide—10 d	Triple therapy—10 d
Andreev et al. [36]	Russia	Triple therapy—10 d; rebamipide—10 d	Triple therapy—10 d
		Triple therapy—10 d; rebamipide—4 w	

**Table 2:** Analysis of selected studies regarding the duration of rebamipide use.

Duration	Studies
Short (10 d–2 w)	Hahm et al. [27]; Nebiki et al. [28]; Lee et al. [31]; Kim et al. [34]; Dicheva et al. [35]; Andreev et al. [36] (1 arm)
Long (4–12 w)	Saita et al. [26]; Kato et al. [29]; Kimura et al. [30]; Fujioka et al. [32]; Simanenkov et al. [33]; Andreev et al. [36] (2 arms)

**Table 3:** Analysis of selected studies on the administered regimens with the inclusion of rebamipide.

Rebamipide-Containing Regimen	Studies
Dual therapy (PPI + amoxicillin)	Saita et al. [26]; Hahm et al. [27]; Nebiki et al. [28]; Kato et al. [29]; Fujioka et al. [32];
Triple therapy (PPI + amoxicillin + clarithromycin/metronidazole)	Kimura et al. [30]; Lee et al. [31]; Dicheva et al. [35]; Andreev et al. [36]
Triple therapy with bismuth (PPI + amoxicillin + clarithromycin + bismuth tripotassium dicitrate)	Simanenkov et al. [33]
Concomitant therapy (PPI + amoxicillin + clarithromycin + metronidazole)	Kim et al. [34]

## Supplementary Materials

The following are available online at https://www.mdpi.com/2077-0383/8/9/1498/s1: Table S1. The results of the statistical analysis of the selected studies.

## References

[1] Suzuki H., Warren R., Marshall B. (2016). Helicobacter Pylori.

[2] Hooi J.K.Y., Lai W.Y., Ng W.K., Suen M.M.Y., Underwood F.E., Tanyingoh D., Malfertheiner P., Graham D.Y., Wong V.W.S., Wu J.C.Y. (2017). Global prevalence of *Helicobacter pylori* infection: Systematic review and meta-analysis. Gastroenterology.

[3] Malfertheiner P., Link A., Selgrad M. (2014). *Helicobacter pylori*: Perspectives and time trends. Nat. Rev. Gastroenterol. Hepatol..

[4] Peek R.M., Crabtree J.E. (2006). Helicobacter infection and gastric neoplasia. J. Pathol..

[5] Malfertheiner P., Megraud F., O’Morain C.A., Gisbert J.P., Kuipers E.J., Axon A.T., Bazzoli F., Gasbarrini A., Atherton J., Graham D.Y. (2017). Management of *Helicobacter pylori* infection-the maastricht V/Florence consensus report. Gut.

[6] Fallone C.A., Chiba N., van Zanten S.V., Fischbach L., Gisbert J.P., Hunt R.H., Jones N.L., Render C., Leontiadis G.I., Moayyedi P. (2016). The Toronto consensus for the treatment of *Helicobacter pylori* infection in adults. Gastroenterology.

[7] Chey W.D., Leontiadis G.I., Howden C.W., Moss S.F. (2017). ACG clinical guideline: Treatment of *Helicobacter pylori* infection. Am. J. Gastroenterol..

[8] Zhou L., Sung J.J., Lin S., Jin Z., Ding S., Huang X., Xia Z., Guo H., Liu J., Chao W. (2003). A five-year follow-up study on the pathological changes of gastric mucosa after *H. pylori* eradication. Chin. Med. J..

[9] Okubo M., Tahara T., Shibata T., Nakamura M., Yoshioka D., Maeda Y., Yonemura J., Ishizuka T., Arisawa T., Hirata I. (2011). Changes in gastric mucosal patterns seen by magnifying NBI during *H. pylori* eradication. J. Gastroenterol..

[10] Lee Y.C., Chen T.H., Chiu H.M., Shun C.T., Chiang H., Liu T.Y., Wu M.S., Lin J.T. (2013). The benefit of mass eradication of *Helicobacter pylori* infection: A community-based study of gastric cancer prevention. Gut.

[11] Safavi M., Sabourian R., Foroumadi A. (2016). Treatment of *Helicobacter pylori* infection: Current and future insights. World J. Clin. Cases.

[12] Feng L., Wen M.Y., Zhu Y.J., Men R.T., Yang L. (2016). Sequential therapy or standard triple therapy for *Helicobacter pylori* infection: An updated systematic review. Am. J. Ther..

[13] Venerito M., Krieger T., Ecker T., Leandro G., Malfertheiner P. (2013). Meta-analysis of bismuth quadruple therapy versus clarithromycin triple therapy for empiric primary treatment of *Helicobacter pylori* infection. Digestion.

[14] Puig I., Baylina M., Sanchez-Delgado J., Lopez-Gongora S., Suarez D., Garcia-Iglesias P., Munoz N., Gisbert J.P., Dacoll C., Cohen H. (2016). Systematic review and meta-analysis: Triple therapy combining a proton-pump inhibitor, amoxicillin and metronidazole for *Helicobacter pylori* first-line treatment. J. Antimicrob. Chemother..

[15] Gisbert J.P., McNicholl A.G. (2017). Optimization strategies aimed to increase the efficacy of *H. pylori* eradication therapies. Helicobacter.

[16] Andreev D.N., Dicheva D.T., Maev I.V. (2017). Possibilities for optimization of eradication therapy for *Helicobacter pylori* infection in modern clinical practice. Ter. Arkhiv.

[17] Dore M.P., Lu H., Graham D.Y. (2016). Role of bismuth in improving *Helicobacter pylori* eradication with triple therapy. Gut.

[18] Alkim H., Koksal A.R., Boga S., Sen I., Alkim C. (2017). Role of bismuth in the eradication of *Helicobacter pylori*. Am. J. Ther..

[19] Wang F., Feng J., Chen P., Liu X., Ma M., Zhou R., Chang Y., Liu J., Li J., Zhao Q. (2017). Probiotics in *Helicobacter pylori* eradication therapy: Systematic review and network meta-analysis. Clin. Res. Hepatol. Gastroenterol..

[20] Zhu X.Y., Liu F. (2017). Probiotics as an adjuvant treatment in *Helicobacter pylori* eradication therapy. J. Dig. Dis..

[21] Hojo M., Miwa H., Kikuchi S., Sato N. (2000). Do mucosal defensive agents improve the cure rate when used with dual or triple therapy regimens for eradicating *Helicobacter pylori* infection?. Aliment. Pharmacol. Ther..

[22] Naito Y., Yoshikawa T. (2010). Rebamipide: A gastrointestinal protective drug with pleiotropic activities. Expert Rev. Gastroenterol. Hepatol..

[23] Hayashi S., Sugiyama T., Amano K., Isogai H., Isogai E., Aihara M., Kikuchi M., Asaka M., Yokota K., Oguma K. (1998). Effect of rebamipide, a novel antiulcer agent, on *Helicobacter pylori* adhesion to gastric epithelial cells. Antimicrob. Agents Chemother..

[24] Lee K.H., Kim J.Y., Kim W.K., Shin D.H., Choi K.U., Kim D.W., Lee W.J., Choi J.H., Lee S.H., Kim G.H. (2011). Protective effect of rebamipide against *Helicobacter pylori*-CagA-induced effects on gastric epithelial cells. Dig. Dis. Sci..

[25] Nishizawa T., Nishizawa Y., Yahagi N., Kanai T., Takahashi M., Suzuki H. (2014). Effect of supplementation with rebamipide for *Helicobacter pylori* eradication therapy: A systematic review and meta-analysis. J. Gastroenterol. Hepatol..

[26] Saita H., Takahashi Y., Sou Y. (1996). Combination therapy with lansoprazole, amoxicillin, and rabeprazole for cure of *Helicobacter pylori* infection and histlogical gastritis in gastric ulcer patients. Jpn. Arch. Intern..

[27] Hahm K.B., Lee K.J., Kim Y.S., Kim J.H., Cho S.W., Yim H., Joo H.J. (1998). Augmented eradication rates of *Helicobacter pylori* by new combination therapy with lansoprazole, amoxicillin, and rebamipide. Dig. Dis. Sci..

[28] Nebiki H., Higuchi K., Arakawa T., Ando K., Uchida T., Ito H., Harihara S., Kuroki T., Kobayashi K. (1998). Effect of rebamipide on *Helicobacter pylori* infection in patients with peptic ulcer. Dig. Dis. Sci..

[29] Kato M., Asaka M., Sugiyama T., Kudo M., Nishikawa K., Sukegawa M., Hokari K., Katagiri M., Sato F., Kagaya H. (1998). Effects of rebamipide in combination with lansoprazole and amoxicillin on *Helicobacter pylori*-infected gastric ulcer patients. Dig. Dis. Sci..

[30] Kimura M., Urakami Y., Seki H. (1999). Effects of mucosalprotective agents in combinaiton with eradication therapy on *Helicobacter pylori*-infected gastric ulcer. Front. Gastroenterol..

[31] Lee D.S., Ahn B.M., Lee K.M., Jeong H.Y., Lee M.H., Chung I.K., Roe I.H., Nam S.W., Lee J.D. (2000). Effect of rebamipide (Mucosta(R)) in eradication of *Helicobacter pylori*. Korean J. Gastrointest. Endosc..

[32] Fujioka T., Arakawa T., Shimoyama T., Yoshikawa T., Itoh M., Asaka M., Ishii H., Kuwayama H., Sato R., Kawai S. (2003). Effects of rebamipide, a gastro-protective drug on the *Helicobacter pylori* status and inflammation in the gastric mucosa of patients with gastric ulcer: A randomized double-blind placebo-controlled multicentre trial. Aliment. Pharmacol. Ther..

[33] Simanenkov V.I., Bakulina N.V., Fil T.S., Khubieva A.K. (2017). Evaluation of efficiency of *H. pylori* eradication in case of addition of cytoprotective preparation rebamipide to the treatment: Results of the BASTION trial. Farmateka.

[34] Kim J., Kim K., Lee J.S., Kim S.Y., Kim K.O., Kim Y.J., Kwon K.A., Park D.K., Chung J.W. (2018). The efficacy of rebamipide or ecabet sodium supplementation for *Helicobacter pylori* eradication therapy compared with quadruple (concomitant) regimen. Korean J. Gastroenterol..

[35] Dicheva D.T., Andreev D.N., Partsvania-Vinogradova I.V., Maev I.V. (2018). Evaluation of efficacy and safety of rebamipide use in the triple therapy for *Helicobacter pylori* eradication: A pilot study. Med. Counc..

[36] Andreev D.N., Maev I.V., Dicheva D.T., Samsonov A.A., Partzvania-Vinogradova E.V. (2018). Efficacy and safety of the use of rebamipide in the scheme of triple eradication therapy of *Helicobacter pylori* infection: A prospective randomized comparative study. Ter. Arkh..

[37] Thung I., Aramin H., Vavinskaya V., Gupta S., Park J.Y., Crowe S.E., Valasek M.A. (2016). Review article: The global emergence of *Helicobacter pylori* antibiotic resistance. Aliment. Pharmacol. Ther..

[38] Kamada T., Sato M., Tokutomi T., Watanabe T., Murao T., Matsumoto H., Manabe N., Ito M., Tanaka S., Inoue K. (2015). Rebamipide improves chronic inflammation in the lesser curvature of the corpus after *Helicobacter pylori* eradication: A multicenter study. BioMed Res. Int..

