# Supplementary material for: Efficiency of the Inclusion of Rebamipide in the Eradication Therapy for Helicobacter pylori Infection: Meta-Analysis of Randomized Controlled Studies
Source: J Clin Med. 2019 Sep 19;8(9):1498. doi: 10.3390/jcm8091498 (PMC6780189; doi:10.3390/jcm8091498)
Supplement: Supplementary file 1 [file jcm-08-01498-s001.pdf]

Table S1. The results of the statistical analysis of the selected studies.

Study	Intervention	Controls	Odds ratio	95% CI	z	P	Weight (%)	
							Fixed	Random
Saita et al. [26]	25/33	15/27	2.500	0.832 to 7.511			7.17	7.17
Hahm et al. [27]	27/36	12/21	2.250	0.714 to 7.086			6.59	6.59
Nebiki et al. [28]	44/58	31/56	2.535	1.139 to 5.638			13.57	13.57
Kato et al. [29]	26/38	21/45	2.476	1.006 to 6.093			10.70	10.70
Kimura et al. [30]	25/26	24/27	3.125	0.304 to 32.167			1.60	1.60
Lee et al. [31]	56/62	16/20	2.333	0.586 to 9.291			4.54	4.54
Fujioka et al. [32]	53/82	53/78	0.862	0.447 to 1.662			20.12	20.12
Simanenkov et al. [33]	19/20	15/20	6.333	0.667 to 60.165			1.71	1.71
Simanenkov et al. [33] (2)	19/20	17/20	3.353	0.318 to 35.366			1.56	1.56
Kim et al. [34]	77/85	97/118	2.084	0.875 to 4.962			11.52	11.52
Kim et al. [34] (2)	77/85	66/74	1.167	0.415 to 3.280			8.12	8.12
Dicheva et al. [35]	26/30	18/22	1.444	0.319 to 6.543			3.80	3.80
Andreev et al. [36]	27/32	28/34	1.157	0.316 to 4.243			5.14	5.14
Andreev et al. [36] (2)	21/24	28/34	1.500	0.336 to 6.702			3.87	3.87
Total (fixed effects)	522/631	441/596	1.753	1.312 to 2.343	3.797	<0.001	100.00	100.00
Total (random effects)	522/631	441/596	1.740	1.296 to 2.336	3.686	<0.001	100.00	100.00
